# Patient-Reported Outcome Measures and Risk Factors in a Quality Registry: A Basis for More Patient-Centered Diabetes Care in Sweden 

**DOI:** 10.3390/ijerph111212223

**Published:** 2014-11-26

**Authors:** Sixten Borg, Bo Palaszewski, Ulf-G Gerdtham, Fredrik Ödegaard, Pontus Roos, Soffia Gudbjörnsdottir

**Affiliations:** 1 Health Economics Unit, Department of Clinical Sciences in Malmö, Lund University, SE-223 81 Lund, Sweden; E-Mail: Ulf.Gerdtham@nek.lu.se; 2 The Swedish Institute for Health Economics (IHE), Box 2127, SE-220 02 Lund, Sweden; 3 The National Diabetes Register, SE-413 45 Gothenburg, Sweden; E-Mails: bo.palaszewski@vgregion.se (B.P.); soffia.gudbjornsdottir@medic.gu.se (S.G.); 4 Centre of Registers in Region Vastra Gotaland, SE-413 45 Gothenburg, Sweden; 5 Department of Economics, School of Economics and Management, Box 7082, SE-220 07 Lund, Sweden; 6 Ivey Business School, Western University, 1255 Western Road, London ON N6G ON1, Canada; E-Mail: fodegaard@ivey.uwo.ca; 7 Department of Medicine, Sahlgrenska University Hospital, University of Gothenburg, SE-413 45 Gothenburg, Sweden

**Keywords:** patient-reported outcome measures/PROM, item response theory/IRT, risk factors, registry data, diabetes, patient-centered diabetes care, evaluation

## Abstract

Diabetes is one of the chronic diseases that constitute the greatest disease burden in the world. The Swedish National Diabetes Register is an essential part of the diabetes care system. Currently it mainly records clinical outcomes, but here we describe how it has started to collect patient-reported outcome measures, complementing the standard registry data on clinical outcomes as a basis for evaluating diabetes care. Our aims were to develop a questionnaire to measure patient abilities and judgments of their experience of diabetes care, to describe a Swedish diabetes patient sample in terms of their abilities, judgments, and risk factors, and to characterize groups of patients with a need for improvement. Patient abilities and judgments were estimated using item response theory. Analyzing them together with standard risk factors for diabetes comorbidities showed that the different types of data describe different aspects of a patient’s situation. These aspects occasionally overlap, but not in any particularly useful way. They both provide important information to decision makers, and neither is necessarily more relevant than the other. Both should therefore be considered, to achieve a more complete evaluation of diabetes care and to promote person-centered care.

## 1. Introduction

Diabetes is one of the chronic diseases that constitute the greatest disease burden in the whole world [1]. It has a significant impact on daily life, and patients must be engaged in their disease and its treatment. In Sweden about 4%–5% of the population have diabetes [2], and the majority of these (85%–90%) have type 2 diabetes. The Swedish National Diabetes Register (NDR) was founded in 1996 with the purpose of improving diabetes care, reducing morbidity due to diabetes, and enabling comparisons between caregivers of a number of clinical outcome measures [3,4]. Currently, the NDR covers about 350,000 patients, constituting 85% of all Swedish diabetes patients. The registry has become an essential part of the daily routines of healthcare centers across Sweden. It is used as a tool for evaluating and improving diabetes care, but until recently has mainly recorded clinical outcomes only. In this article, we describe how the registry has started to collect patient-reported outcome measures, and how these data have been used together with registry data on clinical outcome measures as a basis for evaluating diabetes care.

The standard way of evaluating diabetes care is to monitor diabetes comorbidity risk factors or biomarkers such as glycated hemoglobin level (HbA_1c_) to keep them within defined treatment targets. While these clinical risk factors are important, they may not capture all relevant aspects of diabetes care evaluation. If the evaluation of diabetes care relies only on clinical outcomes, patient preferences are ignored. This is unreasonable, since health-related quality of life and welfare should naturally play a vital role. In this article, we attempt to add the patient perspective by including measurements of patients’ abilities and patients’ judgments of the diabetes care provided.

Previous research indicates that using clinical outcomes and patients’ abilities and judgments simultaneously could improve evaluation of diabetes care, for example by reducing problems associated with the physician acting as a double agent by attempting to act both in the best interest of the patient, and in the interest of the funder of the healthcare services [5]. These two interests may be in conflict, and in the worst case may make the physician an imperfect agent for the patient. Further, there could be synergistic effects in outcomes. If patients are not fully informed, they tend to rely on their own experience rather than the physician’s advice [6]. Good access to information should improve confidence and thereby reduce gaps between the patient’s own opinion and the physician’s advice. Hence, efforts that would lead to more positive judgment of the information provided by healthcare, presumably through actually improving how information is given to patients, might eventually lead to improved risk factor control. 

Specifically in diabetes, there appear to be barriers to the routine clinical use of data on patients’ abilities and judgments, including the lack of reference points to which these data can be compared, the tendency of low sensitivity to therapeutic change, and the fact that these data are patient ratings of their current health which do not have the same established link to future health outcomes that clinical risk factors have [7,8,9]. However, important aspects influencing living with diabetes include diabetes self-management ability [10], coping in everyday life [11], fear of hypoglycemia [12], and fear of future complications [13]. Furthermore, patients’ beliefs and attitudes about the disease, cultural aspects, social support, co-morbidities, and financial resources have been identified as barriers to effective diabetes self-management [10]. These barriers need to be addressed to improve the quality of diabetes care, as they tend to obstruct self-management [10]. Another important aspect is how patients experience their contacts with their healthcare provider [14]. Diabetes care is responsible for offering patients evidence-based care which respects their preferences (*i.e*. person-centered care) [15]. The American Diabetes Association cites effective self-management and quality of life as essential outcomes to be evaluated as part of diabetes care [15]. Diabetes teams in Sweden believe they work as person-centered teams, but evaluations from different government studies have shown the opposite [16]. Therefore, it seems worthwhile to demonstrate successful collection and use of data on patients’ abilities and judgments together with clinical outcome measures in diabetes.

### 1.1. Objectives

The overall purpose of the present work was to include patient reported outcomes in NDR’s evaluation of diabetes care. We hereby hope to accomplish a more complete evaluation of diabetes care and its effects, in a way suitable for managing individual patients and for comparing healthcare providers (see discussion). There were three specific aims:

- To develop a questionnaire so that patients' abilities and their judgments of diabetes care can be measured on a scale suitable for detecting and quantifying change.
- To describe a Swedish diabetes patient sample in terms of their abilities and judgments together with registry data on their risk factors, and to study associations between different patient abilities, judgments and risk factors.
- To characterize groups of patients with low abilities, low judgments, or high risk factor levels; namely, groups with a need for improvement. The intention here was to illustrate how the questionnaire can be used in practice, and to see what groups could be identified using ad hoc levels of abilities, judgments, or risk factors. In practice, detecting such patients might trigger some kind of response in the form of an intervention. In the following, we refer to these levels as *ad hoc response levels*.

With these three goals fulfilled, we can use both clinical outcomes and patients’ abilities and judgments when we evaluate care. If we were to compare two groups of patients undergoing, for example, different modes of care or different treatments, and one of them appears better, then this would generate hypotheses about why one is more successful than the other. This would then need to be further analyzed to see whether there is some factor to be discovered that explains good and bad outcomes. 

We used item response theory (IRT) in order to obtain values for the abilities and judgments, and to be able to detect changes in these over time (e.g., the effect of an intervention). This method has several advantages when successfully applied; it is used to estimate latent constructs that cannot be observed directly, such as patient abilities. It reduces a multidimensional set of questions down to a single estimate of the latent construct. It also gives an indication on how well the latent construct is estimated. Finally, it is more robust to missing values than using responses to the questions directly.

### 1.2. Previous Work

There are other studies of similar combinations of patient abilities or judgments and registry data. Glenngard* et al.* investigated whether general-population individuals seeking healthcare had made an active choice of healthcare provider, and also sought to identify determinants of this choice [17]. They worked with registry data combined with population survey data, aggregated on the municipal level, including patients’ judgments of primary care in terms of service, information, and access. A patient's judgment of the care might affect the decision to choose another healthcare provider, but we wish to work with this judgment as part of the dialogue between the healthcare staff and the patient; that is, before such a decision is made. A study by Janlov and Rehnberg of persons in the general population seeking healthcare used a combination of registry data and patient-reported judgments of the quality of care [18]. Their aim was to use these data to determine measures of quality of care. Both these studies used aggregated data on the level of the municipality or healthcare provider, but neither focused on the field of diabetes. 

Work similar to ours was presented by Lundstrom* et al.* [19], but their study was concerned with visual disability in cataract surgery patients. Their purpose was to assess and improve an existing questionnaire to obtain a measure of visual disability for measuring the outcomes of cataract surgery. They used the clinical measure of visual acuity to validate their visual disability construct, and overall found their questionnaire to be highly valid and suitable for routine clinical use.

A number of instruments to collect patient-reported outcomes measures have been developed for diabetes to date [20]. Often, generic health-related quality of life instruments have been used together with diabetes questionnaires addressing treatment satisfaction, symptoms, or obstacles related to having diabetes. Our pilot questionnaire was developed by a group of experts, and was inspired by questionnaires that existed at the time, the literature, and clinical expertise. We hope to show that it can be a useful complement to the registration of clinical risk factors in the evaluation of diabetes care.

## 2. Methods

Our conceptual model consists of a set of patient reported outcomes in the form of patient abilities and judgments of diabetes care, and a set of risk factors. We chose to work with a number of abilities and aspects of judgment of the provided diabetes care, that we considered important. 

The patients’ abilities were:

- Diabetes **self-management** or self-care ability (items 1–4): the patients are able to manage their diabetes treatment, or take any other necessary actions, by themselves. Patients with a good ability should be less limited by diabetes, and thus be more able to live the life they desire. This is an important determinant of diabetes-related quality of life [10,11,21,22]. Poor self-management has also been found to be associated with depression [23,24].
- **Sense of security** despite having diabetes (items 23–25). This ability could also be described as being free of diabetes-related worries. This is an important determinant of good health-related quality of life [12,13].
- Ability to carry out **daily activities** such as social activities (items 29–30) and work-related activities (items 26–28). A good ability indicates a well-functioning patient who has overcome any limitations imposed by the disease, which is an important determinant of good health-related quality of life [25,26,27,28]. Further, the ability to carry out work-related activities is a determinant of costs from a societal perspective as it affects costs of lost production due to illness [29].

From the literature [10,30,31,32,33], we know that important judgments of diabetes care are:

- The quality of the **service** provided (items 9–10, 19–22); for example, did the staff give the patient a good reception? Did they understand the patient's situation? This would affect the patient’s confidence in healthcare and their compliance to treatment and lifestyle advice. Good communication should also make healthcare receptive to the individual needs of the patient.
- The quality of the** information** given by the staff (items 11–14). The visit to healthcare should be an important source of diabetes-related information for the patient. Poorly informed patients may do worse, as described above.
- **Access** to physicians and nurses (items 5–8). Is it easy for the patients to get an appointment with their physician or diabetes nurse when they wish? A timely response to any problem that occurs should be beneficial for the patient.
- **Involvement** in decisions regarding diabetes care (items 15–18), for example regarding medical treatment or diabetes self-care. This should build up the patient’s confidence in healthcare and could be a cornerstone of developing their self-management ability.

We developed a patient questionnaire in order to measure these patient-reported outcomes, several of which are listed by the World Health Organization as important factors for a person’s functioning, disability, and health [34].

In addition, we chose to work with a small set of central risk factors, namely HbA_1c_, systolic blood pressure (SBP), and low-density lipoprotein cholesterol level (LDL). These have been shown to be predictive of cardiovascular disease in diabetes patients [8,9], and are associated with specific treatment targets, such as those included in Swedish diabetes guidelines: HbA_1c_ < 52 mmol/mol, SBP < 130 mmHg, and LDL < 2.5 mmol/L [35]. 

The abilities, judgments, and risk factors that we have listed here represent our view of the patients and their situation. Evaluation of diabetes care alternatives (e.g., treatments) based on these outcomes could offer a way to optimize the patients’ outcomes. The outcomes we have listed do not necessarily constitute a complete list of measures worthy of optimizing, but they provide a manageable set of central measures suitable for trying out our ideas. Further development of our work might include additional measures.

### 2.1. Patient Questionnaire

We developed a diabetes-specific questionnaire with a total of 36 items. Thirteen items (1–4, 23–30, 36) were adapted from the DTSQ, Swe-DES-23, Swe-PAID-20, and SF-36 questionnaires to address self-care/self-management, self-perceived health, diabetes-related worries about metabolic control and complications, and daily social and work-related activities [36,37,38,39]. Nine additional items were constructed to address the patient’s judgment of service provided, information given, access to physicians and nurses, and their own involvement in treatment decisions. Organization can vary between healthcare centers, particularly in terms of whether care is provided by physicians or nurses. Therefore these items asked both about physicians and about nurses, making a total of 18 items (items 5–22). Furthermore, five items addressing mobility, self-care, usual activities, pain/discomfort, and anxiety/depression from the EQ-5D form were included as a known reference instrument (items 31–35) [40]. All resulting items have five response levels, except those from EQ-5D which have three levels. All items have ordered response levels. The questionnaire was issued to patients in Swedish, but the descriptions here of the items and response levels are translated into English (see Appendix).

### 2.2. IRT Model

The responses to the patient questionnaire are ordered response levels on a number of questions related to each of the underlying abilities and judgments; for example, “Strongly disagree”, “Agree”, and so on. These were converted to numerical estimates using item response theory [41,42], a technique to estimate an underlying value, or latent variable, which cannot be observed directly. In our case, the patient abilities and judgments are considered latent variables. Items are grouped into scales, each related to one latent variable. IRT uses a scale model of the relationship between the latent variable and the responses to the items. Individual IRT scores are estimated from each patient’s response patterns. The purpose of IRT is to obtain an estimate that has interval properties, so that the difference between two points on the scale gives a meaningful quantity. This is not achieved with raw summary scores based directly on the responses, such as the sum of the response levels that a patient has chosen for a set of items, as summing in this way does not take into account whether a given level on one item is more difficult to endorse than that of another item. IRT allows items, as well as item response levels, to have different difficulties. 

We grouped the items into scales reflecting each of the abilities and judgments listed above. The definition and review of each scale, fitting a scale model, and review of its fit was undertaken according to a number of steps until a final set of scales was obtained. First we used non-parametric IRT in order to check for unidimensionality, local dependency, and latent monotonicity [43]. As another check of unidimensionality, a parallel factor analysis was performed on the item set of each scale [44,45]. Scales indicating the presence of more than one factor were subjected to factor analyses to identify the items belonging to a second factor. 

We then proceeded with parametric IRT using a graded response model (GRM). The choice of a parametric scale model for polytomous items appears somewhat arbitrary, and often different choices produce nearly identical results [44,46]. In a GRM [47], the probability P of a response in category *k* or lower, in item *i*, given the latent variable *z*, is given by log(P/(1 − P)) =* D_i_ (z − E_ik_)*, where *D_i_* is the discriminant parameter and the *E_ik_* are the extremity parameters. For *k* = 5, P is the complement to the other (*k* = 1,2,3,4). The GRM assumes local independence and latent monotonicity. We evaluated manifest monotonicity, which is a pragmatically valid test for latent monotonicity [43]. Next, GRMs were fitted and tested for equal discrimination [41]. The fit of items was evaluated with S − X^2^ [48], and by comparing probabilities of endorsing an item under its scale model to the observed proportions, since the risk of wrongly flagging items for misfit increases with the number of observations, especially in scales with few items [49]. Overall model fit was evaluated using the root mean square error of approximation (RMSEA) index based on the M_2_ statistic [50]. Hooper* et al.* present a review of various rules of thumb for the magnitude of RMSEA [51], and Milfont and Fischer present similar figures [52]. We adapted this by accepting a point estimate of RMSEA ≤ 0.10 as a fair fit. We examined differential item functioning [41] (*i.e*. whether items are equivalent in meaning to different respondents) between diabetes types and between young and old patients split by median age. We also examined whether any differential item functioning had meaningful impact [41,44]. 

For the judgment of care, we began with separate scales for nurses and for physicians. Depending on how care is organized at a healthcare center, either or both could be relevant. From the patients’ point of view, however, it might not matter whether care is provided by physicians or nurses, and so we also constructed combined scales by taking, one item at a time, the least desirable of the patient's responses regarding nurses and physicians respectively. 

Items 1–30 and 36 were recoded so that the value 1 represented the least desirable response level for the patient, and higher values represented more desirable response levels. The ltm package was used to obtain IRT scores from complete or partially complete response data. Latent variables for each respondent were estimated, and we subsequently transformed them to a scale from 0 (least desirable) to 100 (most desirable). 

Another diabetes patient sample from NDR was used for validation of our IRT approach. We fitted the same GRMs to the responses in the second sample, and used these GRMs to estimate *new IRT scores* using the response patterns in the main sample. These were then compared to the *originally estimated IRT scores*. The correlations and pairwise differences between the new and original scores on each of the abilities and judgments were used to study their agreement.

### 2.3. Methods for Analyzing Outcome Measures

The responses to the EQ-5D questions (31–35) were used to estimate quality-adjusted life-year (QALY) weights—that is, the EQ-5D Index—using a Swedish tariff [53]. 

The latent variables, the EQ-5D Index, the risk factors, and other registry data in the patient sample are described using summary statistics. In order to explore how IRT scores vary with other patient characteristics, we compared groups using the Kolmogorov-Smirnoff test of the empirical distribution functions (EDF) of IRT scores (null hypothesis identical distributions, against different distributions; two-sided). The groups in the tests were men* versus* women, and patients who responded poor* versus* excellent to self-rated health in general (item 36). For risk factors, age, and diabetes duration, we compared the groups of patients below the first quartile* versus* above the third quartile. Correlations between IRT scores, patient covariates, and risk factors were estimated using Spearman’s rho. A *p*-value of <0.0001 was considered to indicate a significant difference in distribution of IRT scores (adjusted for mass significance). We also carried out tests for correlations significantly different from zero. The test on EDFs and the correlations measure different concepts, a test on EDFs detects difference in distribution, for example different shape, scale, or location.

In our analysis of ad hoc response levels, we tested if low levels of abilities or judgments were associated with deviations in risk factors, or if high risk factor levels were associated with deviations in abilities and judgments, compared to the overall sample, in order to see whether some of the collected data might be redundant. Subgroups of patients were defined as the patients with a value less than the tenth percentile of each of the abilities and judgments. Further subgroups were defined as the patients with a value above the ninetieth percentile of each risk factor. If the empirical distribution functions of an IRT score in one such subgroup differed from the overall sample (by diabetes type) with a *p*-value < 0.05, we flagged it as an association. We used this significance level only as a flagging device to identify potential associations that might be worthy of future investigation. 

The review of scales, fitting of GRM models, estimation of latent variables, and further analyses were carried out using software written in the R language [54], namely the mokken [43], ltm [55], and psych [45] packages, our own R code, and IRT Pro [56].

## 3. Material

The NDR has been carrying out projects since 2003 to monitor and improve the quality of diabetes care in Sweden. One such project started in February 2008 and ended in November 2009. Primary care centers and hospital out-patient clinics connected to the NDR were invited to participate, and the first 26 to accept the invitation were included [57,58]. From each of the participating health care centers, we selected all diabetes patients who had visited the center between 1 January and 30 May 2008, and who were aged 18 to 80 years, not recently diagnosed with diabetes (<6 months ago), alive, and not living with protected identity. This was our main sample. In total, 4760 patients were selected for inclusion. The patient sample was representative for the patient population covered by the NDR, and is presented in greater detail elsewhere [57,58]. The questionnaire was sent by mail to the selected patients between June and August 2008, and a reminder was sent to non-responders after two months. A total of 2916 patients (61%) responded and were included in the analysis (Table 1). Of these, 1124 had type 1 diabetes and 1792 had type 2 diabetes, with first and third quartiles of various measures as follows: age 38 and 62* versus* 60 and 73 years, diabetes duration 12 and 33* versus* 3 and 13 years, HbA_1c_ 54 and 70* versus* 46 and 61 mmol/mol, LDL 2.02 and 2.97* versus* 2.02 and 3.17 mmol/L, and SBP 120 and 140* versus* 126 and 146 mmHg, respectively.

The NDR keeps longitudinal records of individual patient data entered by healthcare centers. These records include patient characteristics, type and duration of diabetes, risk factors, diabetes treatment, and more. Each person's data are associated with a unique identification number, which enables registry data to be deterministically linked on the individual level with patient-reported outcomes measures. For our study patients, risk factors were extracted from the NDR together with patient covariates such as age, gender, diabetes type and duration, and diabetes treatment, by extracting the most recent record in the NDR from the one-year period before issuing the questionnaire. Almost all patients (98%) had values for HbA_1c_ and systolic blood pressure, and about 77% of type 1 diabetes patients and 65% of type 2 patients had a value for LDL cholesterol. The availability of values was roughly stable between treatment groups.

**Table 1:** Patient sample characteristics by diabetes type in the main study sample and in the validation sample.

	Main Study Sample		Validation Sample	
	Type 1 diabetes	Type 2 diabetes	Type 1 diabetes	Type 2 diabetes
Age, years	49 (15, 18–80)	66 (10, 21–80)	50 (16, 18–90)	66 (12, 21–95)
Age at diagnosis, years	25 (16, 0–76)	56 (11, 5–79)	26 (17, 0–81)	54 (14, 5–89)
Diabetes duration, years	24 (15, 0–66)	10 (8, 0–54)	24 (15, 0–71)	12 (10, 0–71)
Male / female	50% / 50%	56% / 44%	50% / 50%	57% / 43%
HbA_1c_, mmol/mol	63 (14, 32–136)	55 (14, 26–139)	63 (13, 27–124)	58 (14, 28–133)
Systolic blood pressure, mmHg	128 (15, 90–185)	137 (17, 85–210)	127 (16, 85–200)	134 (16, 80–206)
LDL cholesterol, mmol/l	2.5 (0.8, 0.7–5.2)	2.6 (0.9, 0.5–6.6)	2.7 (0.7, 0.5–6.2)	2.6 (0.9, 0.5–6.2)
Number of patients	1 124	1 792	1 656	1 431

Notes: presented as mean (SD, min–max), or percent (%) of patients.

After the first 26 centers, 12 additional centers participated in a subsequent NDR project in which the same questionnaire was used [59]. A validation sample was formed from this subsequent project, with questionnaire data and registry data extracted as described above. This sample consisted of 1656 type 1 diabetes patients and 1431 type 2 patients (Table 1), corresponding to a response rate of 60%.

Ethical approval for this study was given by the Regional Ethical Review Board of Gothenburg, Sweden.

## 4. Results

### 4.1. IRT Scales

Self-management had to be split into two scales, with knowledge and skills-related items in one scale, and satisfaction and stress-related self-management items in another scale. Item 23 showed local dependence and was removed from the Sense of Security scale. The other scales remained as initially constructed (items 1–30, below). As expected, given the clinical differences between type 1 and 2 diabetes, separate scale models had to be fitted for type 1 and type 2 diabetes. Item 36, self-reported health in general, was not used in any scale and was instead used ad hoc as a stratification variable. The remaining items (31–35) were used to determine the EQ-5D Index. See Appendix for details of the review, and the validation of scales.

The final scales were as follows:

- **Self-Management Skills **(items 1,2): the patient's ability to self-manage diabetes.
- **Self-Management Ability** (3,4): the patient's emotional ability to cope with diabetes.
- **Sense of Security** (24, 25): the patient’s lack of worries related to diabetes comorbidities.
- **Social Activities** (29, 30): the patient’s ability to carry out social activities.
- **Work Activities** (26–28): the patient’s ability to carry out work-related activities.
- **Access** (5–8): the patient’s experience of access to healthcare staff.
- **Service & Information** (9–14, 19–22): the patient’s experience of healthcare service and information provided.
- **Involvement** (15–18): the patient’s judgment of how well they could participate in decisions regarding diabetes treatment and care.

**Figure 1:**
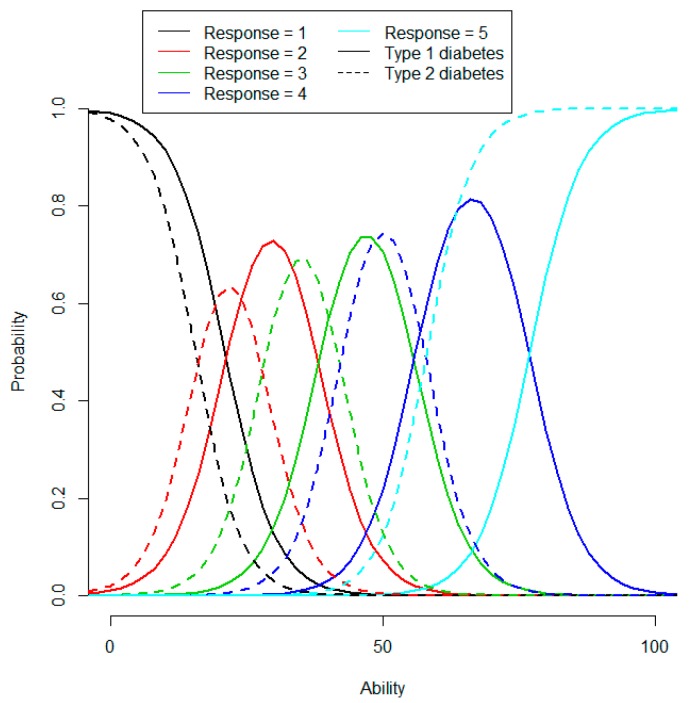
Item response category characteristics curves for item 24 of the Sense of Security scale, by diabetes type. The item is: *During the preceding month, how often have you been worried about having a too low blood sugar level?*

Note: The curves indicate the probability of a patient’s endorsing each level of response (1 = most of the time, ..., 5 = never), for a given ability level.

The item response category characteristic curves for item 24 (Sense of Security) are shown in Figure 1 for type 1 and 2 diabetes. Values of the latent variables are not directly comparable between diabetes types due to separate scale models, and the numerical difference in IRT score that stems from an item due to differential item functioning (the horizontal shift in Figure 1) may or may not correspond to a difference in the actual ability.

**Figure 2:**
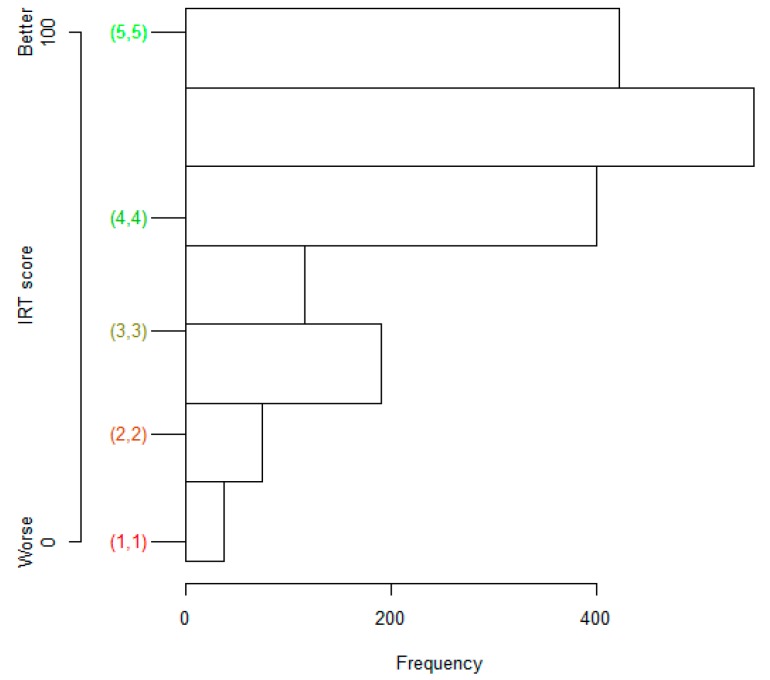
Item-person map for the Sense of Security scale in type 2 diabetes. IRT scores for response patterns **(1,1)** to **(5,5)** are on the left, and a histogram of IRT scores is on the right.

Notes: Item 24 = “During the preceding month, how often have you been worried about having a too low blood sugar level?” Item 25 = “Does your diabetes in general cause you to be worried about the future and the risk of complications?” (1 = Most of time time, 5 = Never).

Our scales do not have any natural reference points, so there will be no absolute reference levels for the latent variables (see Discussion). However, based on the phrasing of the items and their response levels, an item-person map can give meaning to certain ability levels (Figure 2). Further, a unit on a given IRT scale has the same meaning across the whole scale, but a unit on another IRT scale may have a different meaning. Hence, for example, we could say that a given ability is greater in one patient than in another, but we cannot say if the value of any given ability is different from the value of any other ability.

### 4.2. Patient Abilities and Judgments, EQ-5D Index, and Some Determinants

**Figure 3:**
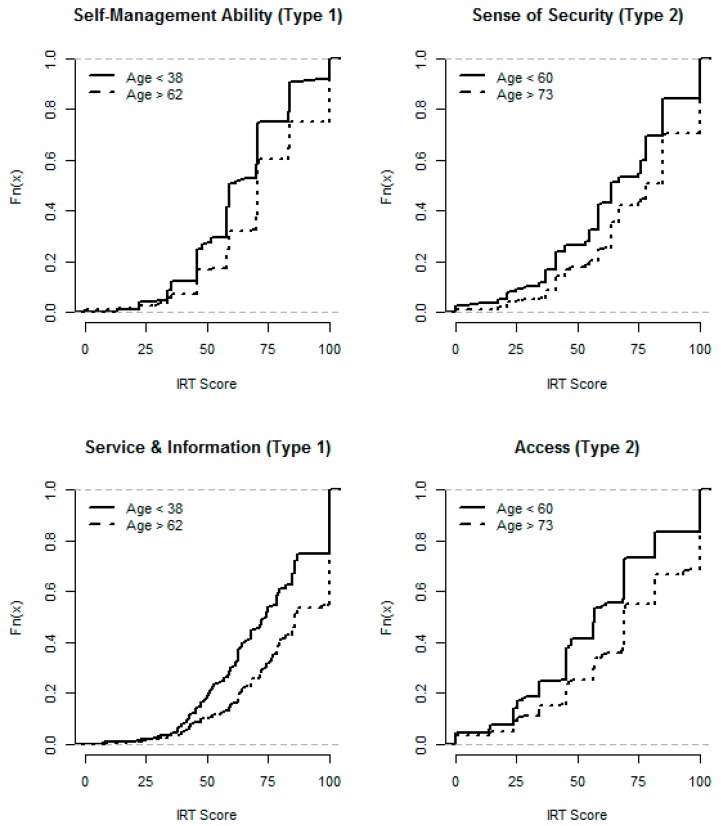
Empirical distribution functions: Self-Management Ability and Service & Information in type 1 diabetes, and Sense of Security and Access in type 2 diabetes, by age groups.

Notes: The distance between the curves shows how the scores differ between the age groups. Empirical distribution function Fn(x) = probability of ( IRT score ≤ x ), so a curve more to the left indicates lower IRT scores. Age groups defined as below the first quartile, and above the third quartile, respectively.

Some of the IRT scores varied by demographic characteristics according to EDF significance tests. Younger patients with type 1 diabetes had lower Self-Management Ability and Service & Information with EDF curves more to the left (Figure 3), and lower Access (data now shown). In type 2 diabetes, younger patients had lower Sense of Security and Access (Figure 3), and lower Self-Management Ability and Service & Information (data now shown). Neither gender nor diabetes duration had any influence on any of the IRT scores. Better self-rated health in general was associated with higher values of every IRT score, in both diabetes types, with the exception of Access in type 2 diabetes. 

**Figure 4:**
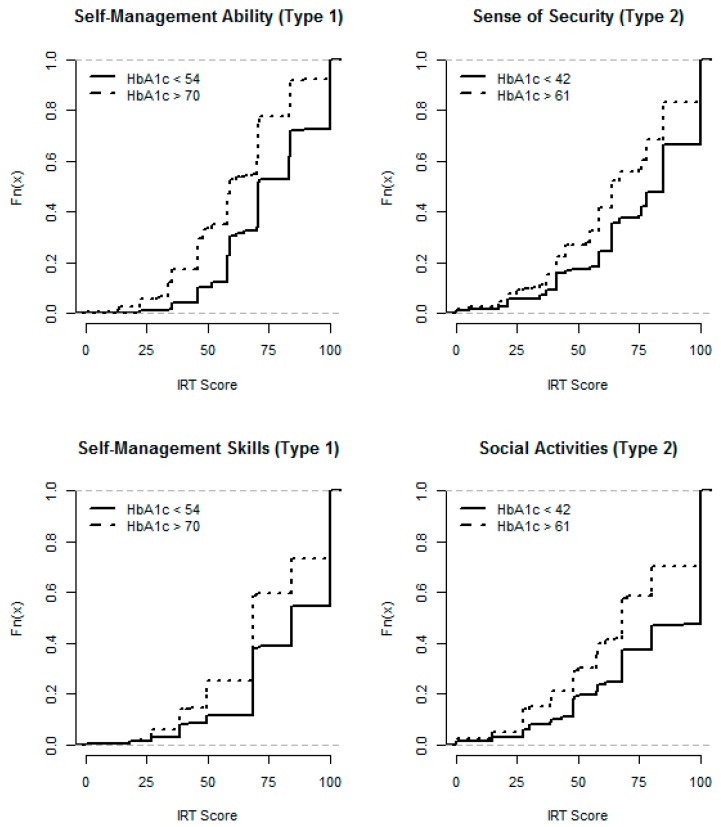
Empirical distribution functions: Self-Management Ability and Skills in type 1 diabetes, and Sense of Security and Social Activities in type 2 diabetes, by HbA_1c_ groups.

Notes: The distance between the curves shows how the scores differ between the HbA_1c_ groups. Empirical distribution function Fn(x) = probability of (IRT Score ≤ x ), so a curve more to the left indicates lower IRT scores. HbA_1c_ groups defined as below the first quartile, and above the third quartile, respectively.

With regard to risk factors, patients with high HbA_1c_ had lower Self-Management Skills and Self-Management Ability in type 1 diabetes, whereas in type 2 diabetes, a high HbA_1c_ was associated with lower Sense of Security and Social Activities (Figure 4, Table 2). Neither SBP nor LDL had any influence on any IRT score, except that SBP was positively correlated with Self-Management Ability in type 1 diabetes (Table 2). The correlations indicated broadly the same associations as the EDF tests between risk factors, abilities and judgments, with the above exception and that HbA_1c_ was negatively correlated with Self-Management Skills and Ability in type 2 diabetes (Table 2).

Men had higher EQ-5D Index than women in both type 1 and 2 diabetes. Type 1 patients with lower age or with a shorter diabetes duration had higher EQ-5D Index than the overall sample. In both diabetes types, a better self-rated health in general was associated with a higher EQ-5D Index. In type 1 diabetes, those with high HbA_1c_ had lower EQ-5D Index, and both low SBP and high SBP were associated with lower EQ-5D Index than overall. The correlation between SBP and EQ-5D Index was not significant (Table 2). The EQ-5D Index was positively correlated with all patient abilities and judgments except Access in type 1 diabetes.

Thus, patient abilities and judgments were mainly associated with self-reported health in general and the EQ-5D Index, to some extent with age, but they were only occasionally associated with risk factors. Further, among the abilities and judgments, the correlations were relatively weak: at most 0.62 between abilities and 0.70 between judgments in type 1 diabetes, and at most 0.73 between abilities and 0.76 between judgments in type 2 diabetes (Table 2). The highest correlation between a risk factor and an abilities or judgment was 0.26 (*i.e*. quite weak).

### 4.3. Analysis of Ad Hoc Response Levels

We examined whether low levels of abilities and judgments were associated with different risk factors levels than in the overall sample. In type 1 diabetes, patients with low Self-Management Skills or low Social Activity had higher HbA_1c_ than the overall average, and patients with low Self-Management Ability had higher HbA_1c_ and lower SBP. In type 2 diabetes, patients with low Sense of Security had higher HbA_1c_, and those with low Self-Management Ability or low Service & Information had lower SBP. No association was seen between low IRT scores and deviations in LDL. 

We then examined whether high risk factor levels were associated with different abilities or judgments than in the overall sample. In type 1 diabetes, high HbA_1c_ values were associated with lower Self-Management Skills, Self-Management Ability, Sense of Security, and Involvement, than the overall average. In type 2, high HbA_1c_ values were associated with lower Self-Management Ability, Sense of Security, Social Activity, Work Activity, and Service & Information than the overall average. There was no association between high LDL and deviations in IRT scores, nor between high SBP and deviations in IRT scores.

**Table 2:** Correlations (Spearman’s rho) between latent variables, risk factors, and EQ-5D Index.

	S-M Skills	S-M Ability	Sense of Security	Social Activities	Work Activities	Access	Service & Infomation	Involvement	HbA_1c_	LDL	SBP	EQ-5D Index
(a) Type 1 diabetes.												
S-M Skills	1.00 *											
S-M Ability	0.60 *	1.00 *										
Sense of Security	0.33 *	0.48 *	1.00 *									
Social Activities	0.30 *	0.43 *	0.48 *	1.00 *								
Work Activities	0.26 *	0.40 *	0.43 *	0.62 *	1.00 *							
Access	0.18 *	0.32 *	0.25 *	0.14 *	0.12	1.00 *						
0.37 *	0.41 *	0.32 *	0.27 *	0.21 *	0.55 *	1.00 *						
Involvement	0.44 *	0.39 *	0.27 *	0.25 *	0.19 *	0.41 *	0.70 *	1.00 *				
HbA_1c_	−0.17 *	−0.26 *	−0.11	−0.08	−0.08	−0.10	−0.06	−0.09	1.00 *			
LDL	−0.02	−0.02	−0.03	−0.07	−0.08	−0.04	−0.02	−0.00	0.13 *	1.00 *		
SBP	0.06	0.13 *	0.06	0.04	0.05	−0.00	0.02	0.04	0.04	0.04	1.00 *	
EQ-5D Index	0.21 *	0.34 *	0.37 *	0.06	0.14 *	0.13 *	0.42 *	0.38 *	−0.13 *	−0.04	−0.09	1.00 *
(b) Type 2 diabetes.												
S-M Skills	1.00 *											
S-M Ability	0.73 *	1.00 *										
Sense of Security	0.40 *	0.49 *	1.00 *									
Social Activities	0.36 *	0.46 *	0.54 *	1.00 *								
Work Activities	0.33 *	0.42 *	0.48 *	0.73 *	1.00 *							
Access	0.38 *	0.44 *	0.33 *	0.26 *	0.28 *	1.00 *						
0.47 *	0.51 *	0.34 *	0.32 *	0.32 *	0.68 *	1.00 *						
Involvement	0.51 *	0.49 *	0.31 *	0.28 *	0.27 *	0.58 *	0.76 *	1.00 *				
HbA_1c_	−0.10 *	−0.11 *	−0.14 *	−0.04	−0.04	−0.03	−0.15 *	−0.11	1.00 *			
LDL	−0.03	−0.04	0.01	−0.02	−0.04	−0.00	0.01	0.06	−0.04	1.00 *		
SBP	0.06	0.08	0.06	0.05	0.02	−0.00	0.03	−0.01	0.08	0.06	1.00 *	
EQ-5D Index	0.24 *	0.32 *	0.31 *	0.24 *	0.22 *	0.20 *	0.42 *	0.49 *	−0.06	0.04	0.06	1.00 *

Notes: S-M = Self-management, * = significant correlation, *p* < 0.0001.

## 5. Discussion

We have developed a questionnaire to collect patient abilities and judgments of their experience with diabetes care. The questionnaire was used in a large sample of diabetes patients. We have applied IRT on the data to derive scales for these abilities and judgments. Most of the scales were found to be adequate, and they validated well across two different samples of diabetes patients. Thus we can now use our questionnaire to measure these abilities and judgments in the form of IRT scores.

Secondly, we analyzed these IRT scores together with the patients’ demographic characteristics and risk factor levels. Some IRT scores varied by age; for example, younger patients reported worse experience with diabetes care, and worse Self-Management Ability. On the other hand, gender and diabetes duration did not appear to be determinants of the IRT scores. Of the risk factors, HbA_1c_ was the main factor to show associations with IRT scores; patients with high HbA_1c_ reported poor Self-Management Skills and Ability in type 1 diabetes, and poor Sense of Security and Social Activities in type 2 diabetes. 

We further characterized subgroups of patients with a need for improvement to illustrate practical use of the questionnaire, and also examined whether low levels of abilities and judgments were associated with different risk factor levels than in the overall sample. In type 1 diabetes, for example, we saw that patients with Self-Management Skills had higher HbA_1c_ than the overall sample, and poor Self-Management Ability was associated with low SBP. However, the causal relationship could be in any direction; we cannot say if patients rated their skills poorly because their high HbA_1c_ was obviously a problem, or whether their HbA_1c_ was high because of poor Self-Management skills. In type 2 diabetes, patients with low Sense of Security had higher HbA_1c_, but again, the causal relationship was uncertain. We found no evidence that patients with low IRT scores differed from the overall sample with regards to LDL. When we looked at this in the other direction, to see if high risk factor levels were associated with different levels of abilities and judgments, we only saw associations with HbA_1c_; for example, a high HbA_1c_ level was associated with poor Self-Management Ability and poor Sense of Security in both diabetes types. We found no evidence that patients with high LDL or patients with high SBP differed from the overall sample with regards to IRT scores. High LDL and high SBP often give no symptoms, unlike high HbA1c levels which negatively affects how you feel, so the findings appear logical from that perspective. Looking for patterns going both ways, we found that high HbA_1c_, low Self-Management Skills, and low Self-Management Ability were associated in type 1 diabetes, while high HbA_1c_ and low Sense of Security were associated in type 2 diabetes. Overall, it seemed that patients’ abilities and judgments were not generally associated with the risk factors, and when they were, the association differed between diabetes types. One exception was that patients with low Self-Management Ability had lower SBP in both diabetes types. This might indicate that patients treated for high SBP (and who had, as a result, a lower SBP) were more ill and therefore reported a worse ability. Thus, our IRT scores appear to give one picture of a patient’s situation, and the risk factors another picture, with only partial overlap between the two. Risk factors alone will not tell the healthcare provider how a patient is feeling, and thus are poorly sensitive to health-related quality of life. Conversely, a patient could feel well but still have high levels of risk factors, and thus be at risk of avoidable diabetes complications unless risk factors are considered too. We argue that patients’ abilities, judgments and risk factors all provide important information to decision makers; the risk factors are useful for prognoses regarding future outcomes and costs, and the patients’ abilities and judgments reflect patient preferences. The use of both data sources could achieve a more complete evaluation of diabetes care and promote person-centered care.

There are a number of aspects of our study that deserve special attention. One important aspect is the interpretation of the scales and the IRT scores. We cannot make direct comparisons of the latent variables between diabetes types because they have separate scale models. On the other hand, it is logical from a clinical point of view to have separate scales for the two diabetes types, since what is possible to accomplish for a type 1 patient may be very different from what is possible for a type 2 patient. Our scales lack natural (fixed) reference points to which we could assign some reference levels in the same way as, for example, weight, where 0 represents absence of weight. Nonetheless, we have developed a set of scales which can be used to collect data on patients and eventually follow up their outcomes to determine empirical reference levels. We have the data to describe a Swedish population of type 1 and 2 diabetes patients. Our dataset is fairly unique, in that we have data on patient abilities and their judgments of diabetes care deterministically linked with medical outcome measures from a National Diabetes Registry, on the individual patient level, from about three thousand individuals in our main sample and another three thousand in the patient sample used for validation. Taking this as a baseline, we have to date roughly 20,000 patient-years of follow-up.

We cover a number of dimensions judged as important for the diabetes patient. There might be further dimensions that need to be included to make the scope more complete, and work on this is ongoing at the NDR. However, we have now taken this first step of analyzing a combination of patients’ abilities, patients’ judgments, and risk factors. This could be extended in future work, e.g. by assigning weights to these various dimensions to obtain a single measure of value. This would simplify comparisons between e.g. healthcare providers or modes of diabetes care. It would be interesting to determine such weights, for instance by relating the various dimensions to patients' own valuations, or by relating them to registry outcomes later on, like survival or occurrence of complications. Knowing these weights could help prioritizing between interventions on, say, a poor Sense of Security, a high risk factor value, or indeed on both.

We derived the EQ-5D Index, and we asked about self-rated health in general. It could be asked whether one of these measures would be sufficient, as they were both correlated with most of the IRT scores. However, they do not reveal which specific aspect is poor. We argue that our set of IRT scores, linked with registry data on risk factors, offers more detailed and disease-specific information that we can use for studying and improving the diabetes patient's situation, because it captures more dimensions and has a greater ability to measure changes. On the other hand, our approach is disease-specific, so it cannot be used to make comparisons across diseases in the same way as a generic instrument such as the EQ-5D, and this is an important limitation. However, the instruments can easily be used together as we have done, and we do have the EQ-5D Index and self-rated health in general too.

There are a couple of technical details that should be mentioned. We defined subgroups with poor IRT scores and risk factors using arbitrary thresholds. This serves to illustrate the concept of using a level that would trigger some kind of response (*i.e*. an intervention). However, the actual thresholds could be defined differently. Some of our scales have very few items, and this affects their measurement properties and their robustness to missing values. Item 25 in the Feeling Safe scale displayed misfit, but it was kept because the scale contains only two items. New or additional items might be needed, but may conflict with having a short questionnaire that can be filled in without too much effort. Further, our Work Activities scale did not work well in any diabetes type, and requires further development. A revision of the questionnaire is ongoing at NDR. Additional scale properties are discussed in the appendix.

We see several potential implications of our work that should be beneficial. Optimizing patients’ abilities and judgments should result in improving their health-related quality of life, diabetes self-management abilities, and experience of diabetes care. Reasons for poor risk factor control may be found by analyzing patient abilities and judgments, and this can guide caregivers in helping patients reach their treatment targets and thereby reduce their risk of future complications. In connection to a healthcare visit, estimates of a patient's abilities could be obtained and used in the dialogue between the physician and the patient along with the risk factors, to decide on further treatment, care or support. Judgments of the provided care might be a bit sensitive in a face-to-face meeting. But on the healthcare provider level, judgments as well as abilities and risk factors could be used to compare healthcare production between providers or between diabetes care alternatives, since the patient's judgments of their experience with diabetes care ought to be useful intermediate endpoints in optimizing the interaction between the patient and the healthcare provider.

We believe there is a strong health-economic value here that translates into avoided morbidity and thereby avoided costs for society and avoided burden for the patients. 

## 6. Conclusions

We have successfully collected data to estimate patient-reported outcome measures in the form of patient abilities and judgments of their experience of diabetes care. Analysis of these together with risk factors showed that the different types of data describe different aspects of a patient's situation. These aspects occasionally overlap, but not in any particularly useful way. They both provide important information to decision makers and none is necessarily more relevant than the other. Both should therefore be considered, to achieve a more complete evaluation of diabetes care and to promote person-centered care.

## Appendix

### A1. IRT Analysis

None of the assumptions for non-parametric IRT were violated by the final scales, nor did these scales show any signs of presence of more than one factor in the parallel factor analyses. In the parametric IRT, the scale model coefficients showed no anomalies or violations of the assumption of local independence and the assumption of unidimensionality. Item 23 was flagged for local dependence, and was removed. Many items were flagged for misfit by S − X^2^, probably due to the high number of respondents. Using graphical methods, most items were judged to show adequate fit, but item 25 showed some minor misfit in Feeling Secure for type 2 diabetes. As this scale had very few items, we decided to keep the item. The Self-Management scale demonstrated poor overall fit using RMSEA. However, after self-management knowledge and skills-related items were placed in one scale, and satisfaction and stress-related self-management items were placed in another scale, overall fit improved. M_2_ indicated a poor overall scale model fit for all scales, but RMSEA indicated a fair fit in all scales except Work Activity for both diabetes types. The Self-Management Skills and Self-Management Ability scales just barely indicated a fair fit for type 2 diabetes (Table A1). Differential item functioning was detected with regard to diabetes type, and separate scale models were fitted for each type (Table A2); both types had the same items in every scale, but each diabetes type had its own set of item parameters. For the scales for judgments that had identical pairs of items for nurses and physicians, we selected the combined scales as their overall model fit showed an improvement over the separate nurse and physician scales. The fitted GRMs for the scales are presented in Table A2.

**Table A1:** Summary of scale properties for the final scales: number of complete cases (N) and root mean square error of approximation (RMSEA) with 90% confidence intervals (CI), based on M_2_, by type of diabetes.

Scale	N	No of Items	RMSEA	RMSEA (90% CI)
Type 1 diabetes				
Self-Management Skills	1119	2	0.066	(0.052, 0.079)
Self-Management Ability	1110	2	0.064	(0.050, 0.078)
Sense of Security	1113	2	0.062	(0.047, 0.075)
Social Activities	1110	2	0.062	(0.048, 0.076)
Work Activities	756	3	0.452	(0.444, 0.461)
Access	1073	2	0.063	(0.048, 0.076)
Service & Information	1102	5	0.049	(0.044, 0.053)
Involvement	1098	2	0.077	(0.063, 0.090)
Type 2 diabetes				
Self-Management Skills	1763	2	0.102	(0.091, 0.112)
Self-Management Ability	1759	2	0.092	(0.081, 0.103)
Sense of Security	1765	2	0.055	(0.043, 0.065)
Social Activities	1760	2	0.079	(0.068, 0.089)
Work Activities	690	3	0.893	(0.884, 0.902)
Access	1728	2	0.064	(0.053, 0.075)
Service & Information	1726	5	0.056	(0.052, 0.059)
Involvement	1696	2	0.085	(0.074, 0.095)

### A2. Validation of Scales

The new IRT scores and the originally estimated scores had a correlation of at least 0.93. Further, their mean pairwise differences were less than 2 units (the latent variables ranged from 0 to 100) in all scales except Work Activities in type 2 diabetes (7.4 units), thus presenting no signs of systematic differences except in Work Activities (Table A3).

**Table A2:** Fitted graded response models for type 1 and type 2 diabetes, respectively (discriminant parameter ***D_i_*** and extremity parameters ***E_ik_***), for Self-Management Skills (SM 1), Self-Management Ability (SM 2), Sense of Security (SS), Social Activities (SA), Work Activities (WA), Access (A), Service & Information (SI), and Involvement (I).

	Type 1 Diabetes						Type 2 Diabetes				
Scale	Item	D*_i_*	E*_i1_*	E*_i2_*	E*_i3_*	E*_i4_*	D*_i_*	E*_i1_*	E*_i2_*	E*_i3_*	E*_i4_*
SM 1	1	4.617	−2.654	−2.045	−1.173	0.153	4.248	−2.536	−1.799	−0.736	0.385
	2	4.617	−2.612	−2.029	−1.126	0.257	4.248	−2.398	−1.780	−0.756	0.390
SM 2	3	1.977	−2.718	−1.764	−0.624	0.614	2.370	−2.674	−1.777	−0.781	0.362
	4	1.977	−3.134	−1.859	−0.389	1.008	2.370	−2.683	−1.811	−0.771	0.424
SS	24	1.917	−2.456	−1.347	−0.253	1.071	4.826	−2.218	−1.595	−0.887	−0.094
	25	1.917	−1.974	−0.956	0.066	1.551	1.621	−2.444	−1.402	−0.356	0.843
SA	29	2.913	−1.773	−1.024	−0.228	0.889	4.534	−1.717	−1.026	−0.464	0.257
	30	4.604	−1.901	−1.172	−0.565	0.280	4.534	−1.846	−1.050	−0.522	0.057
WA	26	3.078	−2.078	−1.397	−0.694	0.223	3.497	−2.256	−1.483	−0.888	−0.262
	27	4.969	−2.322	−1.682	−1.080	−0.317	5.037	−2.455	−1.653	−1.053	−0.500
	28	3.783	−2.272	−1.462	−0.891	−0.108	5.006	−2.473	−1.564	−1.012	−0.372
A *****	5&6	3.045	−1.691	−0.974	−0.041	0.789	3.794	−1.664	−1.025	−0.333	0.421
	7&8	3.045	−1.649	−1.000	−0.096	1.009	3.794	−1.703	−1.139	−0.337	0.508
SI *****	9&10	3.645	−2.361	−1.607	−0.877	−0.076	3.740	−2.434	−1.742	−0.978	−0.202
	11&12	2.938	−2.865	−1.988	−1.104	−0.201	3.697	−2.355	−1.677	−1.024	−0.206
	13&14	3.390	−2.318	−1.693	−0.819	0.075	3.070	−2.207	−1.613	−0.861	0.089
	19&20	4.692	−2.434	−1.853	−1.221	−0.597	4.360	−2.414	−1.869	−1.217	−0.556
	21&22	4.910	−2.169	−1.686	−1.046	−0.226	4.927	−2.210	−1.750	−1.035	−0.252
I*	15&16	4.617	−2.445	−1.795	−1.036	−0.142	4.597	−1.773	−1.254	−0.568	0.267
	17&18	4.617	−2.354	−1.698	−0.989	−0.027	4.597	−1.634	−1.174	−0.529	0.292

Notes: ***** The responses to pairs of physician and nurse items of the same type are combined.

**Table A3:** Correlation and pairwise differences between each originally estimated IRT score and the corresponding new IRT score based on a scale model from another patient sample.

Scale	Type 1 Diabetes			Type 2 Diabetes		
	Correlation	Difference *, Mean (SD)		Correlation	Difference *, Mean (SD)	
Self-Management Skills	1.00	−1.4	(1.6)	1.00	0.2	(0.6)
Self-Management Ability	1.00	−0.5	(0.5)	0.96	0.1	(6.2)
Sense of Security	1.00	−0.6	(0.7)	1.00	−1.2	(1.0)
Social Activities	0.99	0.5	(3.2)	1.00	0.6	(0.8)
Work Activities	1.00	−0.7	(1.0)	0.93	7.4	(6.1)
Access	0.97	−1.6	(6.1)	1.00	0.6	(1.0)
Service & Information	0.99	−0.7	(3.4)	0.99	−0.3	(2.9)
Involvement	1.00	0.5	(1.1)	1.00	0.1	(0.7)

***** The latent variables are scaled from 0 to 100.

### A3. Discussion of the Scales

A problem with some of our scales is that they consist either only of negative items (e.g., Sense of Security), or only of positive items (e.g., Self-Management Skills), and so respondents with a tendency to agree (or disagree) with items regardless of content would get very high (or low) scores. This risk would be less with a mixture of items [60], although this would be difficult to accomplish for the Sense of Security scale, which is concerned with the patient's worries. Our scales for Access, Service & Information, and Involvement consist of neutral items, each with both positive and negative response levels. Our sample size appears more than adequate to fit GRM models to our scales and estimate latent variables [42,44]. We have 1124 type 1 and 1792 type 2 diabetes patients providing data for most of our scales. On the other hand, due to large sample size, some methods for detecting misfit reach a very high statistical power. We argue from a pragmatic point of view that they thereby overflag misfit, and we choose other methods instead; for example, we use RMSEA to review overall model fit. It is important that the sample being examined is heterogeneous and representative for the whole target population; otherwise, the results in terms of the fitted scale models may only be valid for a subset of the intended target population [46]. We have validated our scales using another patient sample of about the same size. However, we compare predictions from scale models, so individual variation has in a sense been removed. This will result in higher correlation than would have been found had we had actual observations on the native and alternative latent variables. On the other hand, we see very high correlations, so even when allowing for overestimated the correlations, our validation should hold.
